# Socioeconomic and Contextual Differentials in Memory Decline: A Cross-Country Investigation Between England and China

**DOI:** 10.1093/geronb/gbac163

**Published:** 2023-01-10

**Authors:** Dorina Cadar, Laura Brocklebank, Li Yan, Yaohui Zhao, Andrew Steptoe

**Affiliations:** Centre for Dementia Studies, Department of Neuroscience, Brighton and Sussex Medical School, Sussex, UK; Department of Primary Care, Brighton and Sussex Medical School, Sussex, UK; Department of Behavioural Science and Health, University College London, London, UK; Department of Behavioural Science and Health, University College London, London, UK; National School of Development, Peking University, Beijing, China; National School of Development, Peking University, Beijing, China; Department of Behavioural Science and Health, University College London, London, UK

**Keywords:** Longitudinal methods, Memory decline, Socioeconomic markers, Urbanicity

## Abstract

**Objectives:**

Although cognitive functioning is strongly associated with biological changes in the brain during the aging process, very little is known about the role of sociocultural differentials between the western and eastern parts of the world. We examined the associations between individual socioeconomic markers (e.g., education, household wealth) and contextual levels characteristics (e.g., urbanicity) with memory performance and memory decline over up to 8 years of follow-up in England and China.

**Methods:**

The analytical samples included participants aged 50+ from the English Longitudinal Study of Aging (*n* = 6,687) and the China Health and Retirement Longitudinal Study (*n* = 10,252). Mixed linear models were employed to examine the association between baseline individual socioeconomic markers (education, wealth) and contextual-level characteristics (urbanicity) on the change in memory over time.

**Results:**

Our analyses showed that higher education and wealth were associated with better baseline memory in both England and China. Still, the impact of contextual-level characteristics such as urbanicity differed between the 2 countries. For English individuals, living in a rural area showed an advantage in memory, while the opposite pattern was observed in China. Memory decline appeared to be socioeconomically patterned by higher education, wealth, and urbanicity in China but not in England.

**Discussion:**

Our findings highlight substantial socioeconomic and contextual inequity in memory performance in both England and China, as well as in the rate of memory decline primarily in China. Public health strategies for preventing memory decline should target the socioeconomic gaps at the individual and contextual levels to protect those particularly disadvantaged.

Socioeconomic status (SES) is a key determinant of health, with notable and marked disparities within and between various individuals and regions worldwide (Marmot & Bell, 2016). The health gap has been a constant target for addressing the socioeconomic inequalities at meso, micro, and macro levels in countries such as England, the United States, and China which experienced remarkable expansion and economic growth during the last few decades (McDowell et al., 2007). Considering China’s progress in terms of economic growth and life expectancy over the past 60 years, cognitive decline and dementia have become major public health concerns, featuring among the leading causes of death (Chen et al., 2011; Piketty et al., 2019). A recent meta-analysis of 41 studies with 112,632 community-dwelling Chinese participants showed a higher prevalence of mild cognitive impairment (MCI) and increased regional inequity with women, rural residents, those with low education, and increased loneliness being mostly at risk (Lu et al., 2021).

However, the role of education and other socioeconomic determinants on the rates of cognitive decline in the Pacific-Asian countries is less clear. There are considerable variations between levels of compulsory education across countries and in access to health systems, with cultural differences in lifestyle behaviors that could account for some of the discrepancies observed in cognitive aging (Wilson et al., 2009). A comparative study based on anchoring vignettes (see Author Note 1) from the American Health and Retirement Study (HRS) and China Health and Retirement Longitudinal Study (CHARLS) showed a high level of discrepancy between self-reported subjective cognitive impairment and memory performance among older adults, with Chinese participants reporting lower severity of subjective cognitive impairment, while their American counterparts were attesting a better memory recall (Wu, 2016). Despite health and education explaining some of the heterogeneity in these findings, the study could not conclude that these discrepancies were related to either social comparisons or cultural differences.

Findings from epidemiologic and clinical studies suggest a myriad of biological, behavioral, social, and environmental factors contributing to cognitive decline (Gottesman et al., 2014; Lehert et al., 2015; Rong et al., 2020; Song et al., 2018) or higher risk for cognitive impairment or dementia (Livingston et al., 2020); yet, so many of these factors are intrinsically socioeconomically patterned (Braveman & Gottlieb, 2014). A recent systematic review and meta-analysis showed that education has a significant association with baseline cognitive performance in older adults but no relationship with cognitive change (Seblova et al., 2020). This is supported by the cognitive reserve hypothesis (Stern, 2002), which suggests that education could provide a neuronal buster and increased neuroplasticity that could compensate for some of the consequences of aging, such as neurodegeneration, until a stage is reached at which neurological deterioration can no longer be masked or contained (Song et al., 2018; Stern, 2009). This may also explain the variability of cognitive decline in those affected with dementia (Stern, 2012). Yet, the heterogeneity between studies exploring cognitive decline remains unexplained mainly by factors such as education, the Gini coefficient, and gross domestic product (Seblova et al., 2020). From a life-course perspective, if education in early life builds some defense through the accumulation of cognitive reserve (Richards & Deary, 2005), then midlife engagement in social and leisure types of activities, as well as cognitive stimulation in the workplace, may play a crucial role in altering dementia risk (Almeida-Meza et al., 2021; Kivimäki et al., 2021). Other socioeconomic markers such as occupational status and wealth may also contribute to cognitive reserve and overall cognitive health (Adam et al., 2013), although reverse causation should not be ignored (Pfeffer et al., 2013). In an English study of middle-aged and older adults, the incidence of dementia was related to the level of wealth rather than education, although significant cohort effects were observed, with a more substantial impact of lower wealth on participants born in later years (Cadar et al., 2018).

In addition to individual-level factors such as education and wealth, there are other important inequalities in terms of contextual and geographical characteristics. These relate to issues such as neighborhood quality, the accessibility of healthy food outlets, walkability, recreational areas, access to cultural engagement, and health care. Previous findings from the English Longitudinal Study of Ageing (ELSA) showed that the Index of Multiple Deprivation, an aggregate measure of neighborhood quality in the United Kingdom, was associated with cognitive performance in older age, independent of education or individual-level SES (Lang et al., 2008). Similarly, in a Chinese study, older participants who lived in more affluent neighborhoods with increased employment service, higher SES, more bus lines, or handicap accessibility had slower cognitive decline compared to those living in a less affluent neighborhoods. Furthermore, these effects were more pronounced in rural than urban areas of China (Luo et al., 2019).

Nevertheless, associations with neighborhood factors have been inconsistent. For example, Meyer and colleagues showed that neighborhood SES across areas of Northern California had limited associations with baseline executive function and semantic memory, but individuals with dementia living in higher socioeconomic neighborhoods experienced faster rates of cognitive decline in these domains. Yet, these associations were explained by individual-level factors such as education, ethnicity, and vascular risk factors (Meyer et al., 2017).

Until recently, very little research attention has been paid to socioeconomic risk factors affecting the rates of cognitive decline in contemporary Chinese society compared with Western countries such as England or the United States. Therefore, we aimed to examine comparatively the relative contributions of different socioeconomic markers (*education and wealth*) and contextual-level characteristics (*urbanicity*), as well as their interaction on memory performance and memory decline in two nationally representative cohorts of English and Chinese. We hypothesized that participants with lower levels of education or wealth or living in a rural area would have a lower baseline memory performance and steeper decline over time in both England and China.

## Method

### Study Population ELSA, England

The data were from the ELSA, a large, multidisciplinary study representative of the English population in terms of socioeconomic profile, geographic region, and health characteristics (Steptoe et al., 2013). A total of 11,392 core members were interviewed in the first wave of ELSA in 2002–2003 and reinterviewed for the subsequent waves every 2 years. Comparisons with the national census showed that the baseline ELSA is a nationally representative sample (Marmot et al., 2003). For harmonization purposes, in these analyses, we only used the available ELSA data starting from Wave 5 (2010–2011) to Wave 9 (2018–2019), spanning over 8 to 9 years. Each participant has provided informed consent at each ELSA wave of data collection.

### Study Population CHARLS, China

The second sample was drawn from the CHARLS, a community-based and nationally representative sample of Chinese residents ages 45 and older, providing high-quality data, including assessments of social, economic, and health status (Zhao et al., 2014). The study period covered in CHARLS ranged from Wave 1 (2011–2012), Wave 2 (2013), Wave 3 (2015), to Wave 4 (2018), ensuring up to a 7-year follow-up period. Each CHARLS participant was asked to sign two copies of informed consent at every wave.

### Memory Performance and Change in Memory Over Time

Memory was tested at every ELSA and CHARLS wave with a word list recall in which the participants were presented with 10 common unrelated words and were asked to recall these words immediately and after a 5-min delay (Rafnsson et al., 2022).

ELSA and CHARLS use the word lists developed for HRS, which comprise four different versions so that different lists can be given to the follow-up wave to each participant. The overall mean scores for immediate recall and delayed recall were summed up in an overall memory score at every wave of each study.

### Socioeconomic and Contextual Indicators

Socioeconomic indicators, including individual-level (education and wealth) and contextual area-level characteristics (urban/rural), were measured in each study at baseline (Wave 5—ELSA and Wave 1—CHARLS). Employing a coordinated approach of data harmonization (Hofer & Piccinin, 2009), educational attainment was similarly classified within both cohorts into four categories: no qualification; low level (0–6 years); medium level (7–11 years), and high level of education (12+ years). Wealth was calculated by summing wealth from household property, possessions, housing, investments, savings, artwork, and jewelry, and net of debt and divided in each cohort into quintiles. Urbanicity was classified in urban versus rural areas. For more detail on each of these measures’ derivation, see Supplementary Tables S1 and S2.

### Covariates

Several covariates were considered within each cohort based on previous findings (15) and the best model fit. These were baseline age (centered to 66 in ELSA and 61 in CHARLS), sex, marital status, a history of heart problems (yes/no), diabetes mellitus (yes/no), depressive symptoms (yes/no), alcohol (yes/no), and smoking (yes/no). Being younger, male, married, having no heart problems, diabetes, depressive symptoms, and not smoking or drinking was used as the reference group.

### Statistical Analyses

The analytical subsamples included only data from core members aged 50 and older in each study at their baseline wave (see Supplementary Figures S1 and S2 for the corresponding flow charts). The associations between each socioeconomic marker and memory decline over up to 8-year follow-up from Waves 5 to 9 in ELSA and Waves 1 to 4 in CHARLS were examined by employing a coordinated analysis of linear mixed models (maximum likelihood estimation, unstructured covariance), which accounts for between- and within-subjects variability across repeated measures, taking into consideration that the same individuals’ measures are correlated. A “time” variable was generated to represent the follow-up from Waves 5 to 9 in ELSA and between Waves 1 and 4 in CHARLS. The time in the study has been created for each study to account for the period between waves, and every unit indicates a 1-year increase in follow-up time (range from 0 to 8 years). Memory change was modeled as a linear function of time measured from the baseline wave until the end of the study period. Random effects for the intercept and slope were fitted for each individual, allowing participants to have different scores at baseline and rates of change in memory. The slopes were adjusted for the baseline memory, as the rate of decline might strongly depend on this. Independent analyses were conducted for each marker of SES and memory change over time within each cohort. To test whether memory trajectories differed between participants, we included in the model the SES marker, covariates, time, baseline memory, time × SES marker, and time × covariates. Unstandardized coefficients and 95% confidence intervals (CIs) for baseline memory (intercept) and linear change (slope) were presented for each of the two cohorts from fully adjusted models. Missing observations were assumed to be missing at random (Little & Rubin, 2002), and model assumptions were verified by examining residuals computed from the predicted values. Both linear and quadratic effects were tested, but the linear model showed a better fit based on the Bayesian Information Criteria (Raftery, 1986; Raftery et al., 2012). Baseline cross-sectional sample weights were used for each cohort analysis to ensure that the sample is representative of the general population. Four supplementary analyses were conducted. In the first analysis, we retested the association for each SES marker while we mutually adjusted for the other two markers. The second analysis presented a sex-stratified investigation for education and urbanicity because these two factors showed a significant sex interaction in CHARLS. The third supplementary analysis examined the rates of memory decline in three subset populations samples matched for baseline memory: subset sample 1 (baseline memory scores <9), subset sample 2 (baseline memory scores 10–12), subset sample 3 (baseline memory 13+). The fourth supplementary analysis explored a more detailed categorization of education within each cohort to better understand the country-level differences in each country’s educational system and its relationship with memory change (see Supplementary Material). All analyses were performed using STATA version 16. The manuscript was written following STROBE guidelines.

## Results

The average follow-up period was 3.53 (*SD* = 2.80) in ELSA and 3.05 (*SD* = 2.56) in CHARLS. Demographics and memory scores at baseline and each follow-up wave for the two studies (ELSA and CHARLS) are presented in Table 1 and Supplementary Tables S3 and S4.

**Table 1.** Psychosocial and Demographic Characteristics of the Baseline Samples

	ELSA	CHARLS
	*n* = 6,687	*n* = 10,252
Age		
Mean (*SD*)	66.65 (9.09)	61.27 (7.57)
Sex		
Male	3,047 (45.57%)	4,824 (47.05%)
Female	3,640 (54.43%)	5,428 (52.95%)
Marital status		
Married	4,429 (66.23%)	8,790 (85.74%)
Unmarried	2,258 (33.77%)	1,462 (14.26%)
Education		
No qualification	1,670 (24.97%)	3,207 (31.28%)
Low level	2,120 (31.70%)	4,221 (41.17%)
Medium level	1,620 (24.23%)	2,348 (22.90%)
High level	1,277 (19.10%)	476 (4.65%)
Wealth		
Lowest quintile	1,123 (16.79%)	1,999 (19.50%)
Second lowest	1,362 (20.37%)	2,056 (20.06%)
Third	1,359 (20.32%)	2,035 (19.85%)
Fourth highest	1,399 (20.92%)	2,088 (20.37%)
Fifth highest	1,444 (21.59%)	2,074 (20.23%)
Urbanicity		
Urban	4,837 (72.33%)	2,371 (23.13%)
Rural	1,850 (27.67%)	7,881 (76.87%)
Heart problems		
No	6,010 (89.88%)	8,830 (86.13%)
Yes	677 (10.12%)	1,422 (13.87%)
Diabetes		
No	5,894 (88.14%)	9,582 (93.47%)
Yes	793 (11.86%)	670 (6.53%)
Depressive symptoms		
No	5,749 (85.97%)	7,183 (70.06%)
Yes	938 (14.03%)	3,069 (29.94%)
Alcohol		
Less than daily	5,193 (77.66%)	8,396 (81.9%)
Daily	1,494 (22.34%)	1,856 (18.1%)
Smoking		
No	5,700 (85.24%)	7,204 (70.27%)
Yes	987 (14.76%)	3,048 (29.73%)

*Notes*: CHARLS = China Health and Retirement Longitudinal Study; ELSA = English Longitudinal Study of Ageing.

The mean baseline was 66.65 (*SD* = 9.09) in ELSA and 61.27 (*SD* = 7.57) in CHARLS. There was a balanced number of men and women in each cohort, and most participants were married. A third of the English sample was educated to a low level, while 70% of the Chinese sample were not educated or educated to a low level. About 70% of ELSA participants live in an urban area, while 77% of CHARLS participants live in a rural area. The overall memory performance was higher and relatively stable across all waves in ELSA participants. In contrast, in CHARLS participants, the overall performance was lower and declined steeper across waves (see Supplementary Tables S3 and S4).

**Figure 1.**
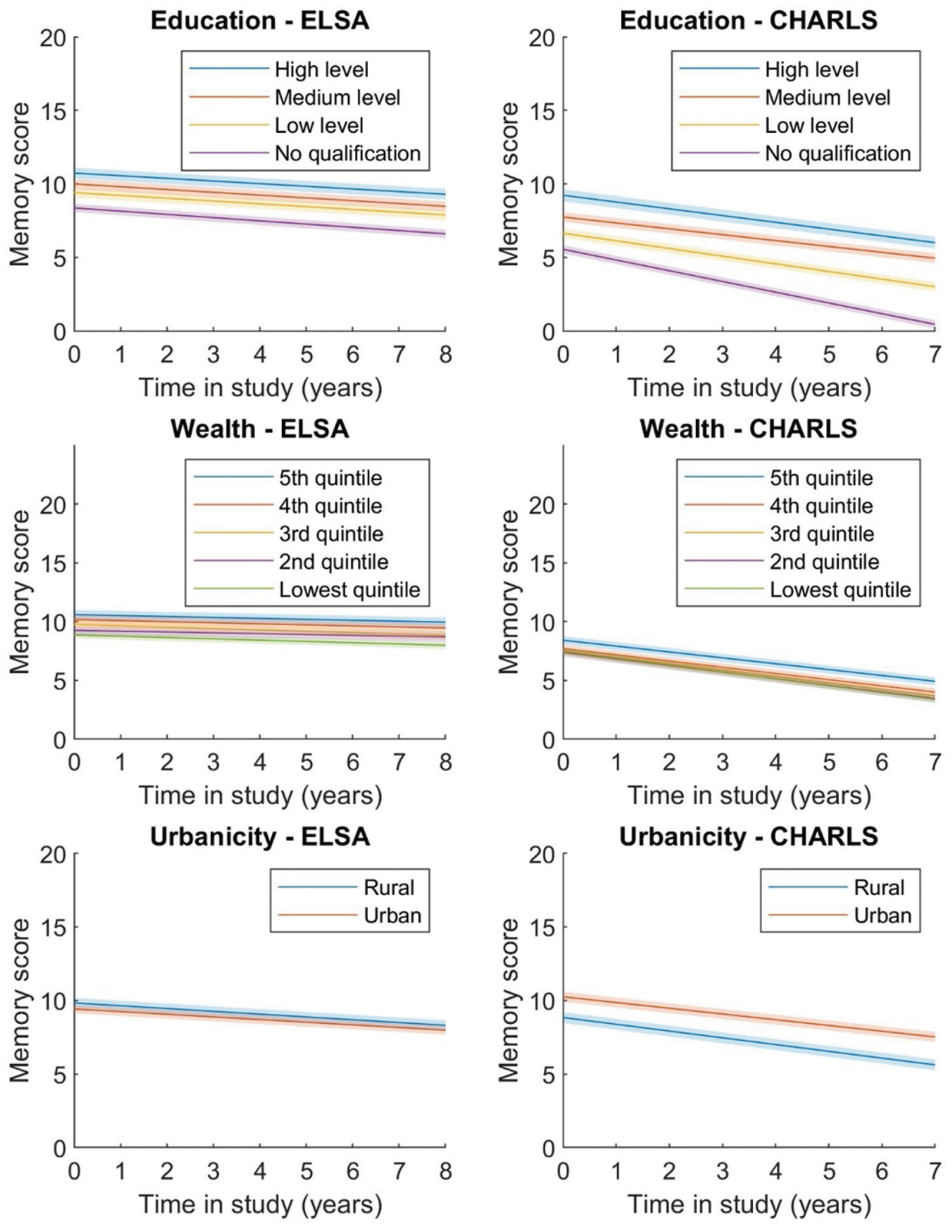
Linear slopes of memory decline by time in the study period, according to each level of socioeconomic markers and urbanicity in fully adjusted analyses* independently coordinated for ELSA and CHARLS. *The models include memory, education, covariates (age centered, sex, marital status, heart problems, diabetes, depressive symptoms, alcohol, smoking), time, time × education, time × baseline memory centered, and time × covariates (age centered, sex, marital status, heart problems, diabetes, depressive symptoms, alcohol, smoking). CHARLS = China Health and Retirement Longitudinal Study; ELSA = English Longitudinal Study of Ageing.

The unstandardized regression coefficients and 95% CIs for the baseline memory (intercept) and linear change (slope) across the two cohorts are shown independently in relation to each socioeconomic marker within each study in fully adjusted models for all the covariates (see Tables 2–4; Figure 1).

The results for education suggested that the reference ELSA participant, an English man of age 66.65, with no formal education, who was married, had no heart problems, diabetes, depressive symptoms, did not drink or smoke, had an average memory of 8.37 (95% CI: 8.08 to 8.66), *p* ≤ .001 and showed an annual decline of −0.22 (95% CI: −0.28 to −0.016), *p* ≤ .001 memory points per year. In comparison with those with no formal education, ELSA participants with higher levels of education showed significant increments in the baseline memory score with 1.04 (95% CI: 0.85 to 1.23), *p* ≤ .001 for low level, 1.63 (95% CI: 1.43 to 1.83), *p* ≤ .001 for medium, and 2.37 (95% CI: 2.13 to 2.60), *p* ≤ .001 for higher levels. No significant associations were observed between higher levels of education and the rate of memory decline in ELSA (see Table 2).

In contrast, the reference CHARLS participant, a Chinese man within the age group of 61.27 years at baseline, with no formal education, who was married, had no heart problems, diabetes, depressive symptoms, did not drink or smoke, had an average memory of 5.56 (95% CI: 5.26 to 5.86), *p* ≤ .001 and showed an annual decline of −0.44 (95% CI: −0.51 to −0.36), *p* ≤ .001 memory points per year. CHARLS participants with higher levels of education also showed significant increments in the baseline memory score with 1.09 (95% CI: 0.95 to 123), *p* ≤ .001 for those with up to low levels, 2.19 (95% CI: 2.02 to 2.35), *p* ≤ .001 for those with medium level, and 3.67 (95% CI: 3.38 to 3.95), *p* ≤ .001 for those with high levels of education. Higher levels of education showed significant protective effects on the rate of memory decline in CHARLS with 0.21 points (95% CI: 0.17 to 0.24), *p* ≤ .001 slower rates of annual memory decline for those with up to low levels, 0.33 (95% CI: 0.29 to 0.37), *p* ≤ .001 for those with medium level, and 0.27 (95% CI: 0.19 to 0.35), *p* ≤ .001 for those with a high level of education (see Table 2 and Figure 1).

**Table 2.** Linear Mixed Models of Education Predicting Memory Over Time in ELSA and CHARLS

	ELSA (*n* = 6,687)	CHARLS (*n* = 10,252)
	Memory	Memory
Initial status	Coefficient (95% CI)	Coefficient (95% CI)
Intercept	8.37 (8.08 to 8.66)***	5.56 (5.26 to 5.86)***
Education		
No qualification	1 (Ref)	1 (Ref)
Low level	1.04 (0.85 to 1.23)***	1.09 (0.95 to 1.23)***
Medium level	1.63 (1.43 to 1.83)***	2.19 (2.02 to 2.35)***
High level	2.37 (2.13 to 2.60)***	3.67 (3.38 to 3.95)***
Rate of linear change	−0.22 (−0.28 to −0.16)***	−0.44 (−0.51 to −0.36)***
Education		
No qualification	1 (Ref)	1 (Ref)
Low level	0.03 (−0.002 to 0.06)	0.21 (0.17 to 0.24)***
Medium level	0.03 (−0.01 to 0.06)	0.33 (0.29 to 0.37)***
High level	0.04 (−0.01 to 0.07)	0.27 (0.19 to 0.35)***
Variance^a^		
Within-person	4.97 (4.79 to 5.15)	6.82 (6.66 to 6.97)
In initial status	4.65 (4.36 to 4.97)	2.96 (2.72 to 3.21)
In rate of change	0.04 (0.02 to 0.06)	0.16 (0.14 to 0.18)
Goodness of fit		
Deviance (−2LL^a^)	−63,682.74	−85,383.98
Wald χ ^2^(29)	3,905.84	8,942.89
*p* Value	≤.001	≤.001

*Notes*: CHARLS = China Health and Retirement Longitudinal Study; CIs = confidence intervals; ELSA = English Longitudinal Study of Ageing; LL = log-likelihood. The models include memory, education, covariates (age centered, sex, marital status, heart problems, diabetes, depressive symptoms, alcohol, smoking), time, time × education, time × baseline memory centered, and time × covariates (age centered, sex, marital status, heart problems, diabetes, depressive symptoms, alcohol, smoking).

^a^The within-person variance is the overall residual variance in memory that is not explained by the model. The initial status variance component is the variance of individuals’ intercepts about the intercept of the average person. Likewise, the rate of change variance component is the variance of individual slopes about the slope of the average person.

***p < .001.

Higher levels of wealth were significantly associated with the baseline memory in ELSA. In comparison to those in the lowest quintile, ELSA participants with higher levels of wealth showed substantially higher baseline memory scores with 0.39 (95% CI: 0.15 to 0.63), *p* ≤ .01 for those in the second lowest quintile, up to 1.73 (95% CI: 1.48 to 1.98), *p* ≤ .001 for those in the highest quintile of wealth. No significant associations were observed between higher levels of wealth and the rate of memory decline in ELSA (see Table 3 and Figure 1).

Similarly, in CHARLS, the top two quintiles of wealth showed a significant association with the intercept, with higher baseline memory scores of 0.26 (95% CI: 0.08 to 0.45), *p* ≤ .01, and 0.98 (95% CI: 0.79 to 1.16), *p* ≤ .001, in comparison with those in the lowest quintile. In relation to memory decline, the highest top quintiles of wealth showed a protective effect with a 0.08 (95% CI: 0.03 to 0.13), *p* ≤ .001 slower change over time (see Table 3 and Figure 1).

**Table 3.** Linear Mixed Models of Household Wealth Predicting Memory Over Time in ELSA and CHARLS

	ELSA (*n* = 6,687)	CHARLS (*n* = 10,252)
	Memory	Memory
Initial status	Coefficient (95% CI)	Coefficient (95% CI)
Intercept	8.86 (8.55 to 9.17)***	7.45 (7.16 to 7.74)***
Wealth		
Lowest quintile	1 (Ref)	1 (Ref)
Second lowest	0.39 (0.15 to 0.63)**	−0.07 (−0.26 to 0.11)
Third	0.88 (0.65 to 1.13)***	0.06 (−0.13 to 0.25)
Fourth highest	1.34 (1.08 to 1.58)***	0.26 (0.08 to 0.45)**
Fifth highest	1.73 (1.48 to 1.98)***	0.98 (0.79 to 1.16)***
Rate of linear change	−0.20 (−0.26 to −0.15)***	−0.17 (−0.25 to −0.10)***
Wealth		
Lowest quintile	1 (Ref)	1 (Ref)
Second lowest	0.04 (−0.003 to 0.08)	0.02 (−0.02 to 0.07)
Third	−0.006 (−0.05 to 0.04)	0.03 (−0.01 to 0.08)
Fourth highest	0.02 (−0.02 to 0.06)	0.05 (−0.02 to 0.11)
Fifth highest	0.03 (−0.02 to 0.07)	0.08 (0.03 to 0.13)***
Variance^a^		
Within-person	4.97 (4.80 to 5.15)	6.81 (6.66 to 6.97)
In initial status	4.91 (4.61 to 5.24)	3.61 (3.35 to 3.87)
In the rate of change	0.04 (0.03 to 0.07)	0.22 (0.19 to 0.25)
Goodness of fit		
Deviance (−2LL^a^)	−63,823.45	−86,582.62
Wald χ ^2^(31)	3,584.07	5,339.59
*p* Value	≤.001	≤.001

*Notes*: CHARLS = China Health and Retirement Longitudinal Study; CIs = confidence intervals; ELSA = English Longitudinal Study of Ageing; LL = log-likelihood. The models include memory, wealth, covariates (age centered, sex, marital status, heart problems, diabetes, depressive symptoms, alcohol, smoking), time, time × wealth, time × baseline memory centered, and time × covariates (age centered, sex, marital status, heart problems, diabetes, depressive symptoms, alcohol, smoking).

^a^The within-person variance is the overall residual variance in memory that is not explained by the model. The initial status variance component is the variance of individuals’ intercepts about the intercept of the average person. Likewise, the rate of change variance component is the variance of individual slopes about the slope of the average person.

**p < .01. ***p < .001.

The results for urbanicity are presented in Table 4. Compared with those living in an urban area, ELSA participants living in rural England showed significantly higher memory scores at baseline with 0.41 (95% CI: 0.25 to 0.57), *p* ≤ .001 but no significant differences in memory decline over time. By contrast, a greater disadvantage was observed for those living in rural China in terms of memory performance with −1.41 (95% CI: −1.55 to −1.27), *p* ≤ .001, and a slightly steeper decline over time with −0.08 (95% CI: −0.12 to −0.04), *p* ≤ .001, compared with those living in an urban area (see Table 4 and Figure 1).

**Table 4.** Linear Mixed Models of Urbanicity Predicting Memory Over Time in ELSA and CHARLS

	ELSA (*n* = 6,687)	CHARLS (*n* = 10,252)
	Memory	Memory
Initial status	Coefficient (95% CI)	Coefficient (95% CI)
Intercept	9.42 (9.10 to 9.73)***	10.25 (9.89 to 10.60)***
Urbanicity		
Urban	1 (Ref)	1 (Ref)
Rural	0.41 (0.25 to 0.57)***	−1.41 (−1.55 to −1.27)***
Rate of linear change	−0.18 (−0.23 to −0.12)*	−0.08 (−0.08 to 0.10)
Urbanicity		
Urban	1 (Ref)	1 (Ref)
Rural	−0.01 (−0.04 to 0.02)	−0.08 (−0.12 to −0.04)***
Variance^a^		
Within-person	4.97 (4.79 to 5.14)	6.81 (6.66 to 6.97)
In initial status	5.20 (4.88 to 5.53)	3.40 (3.15 to 3.67)
In the rate of change	0.04 (0.03 to 0.07)	0.21 (0.19 to 0.24)
Goodness of fit		
Deviance (−2LL^a^)	−63,960.98	−86,317.49
Wald χ ^2^(47)	3,247.98	6,183.64
*p* Value	≤.001	≤.001

*Notes*: CHARLS = China Health and Retirement Longitudinal Study; CIs = confidence intervals; ELSA = English Longitudinal Study of Ageing; LL = log-likelihood. The models include memory, urbanicity, covariates (age centered, sex, marital status, heart problems, diabetes, depressive symptoms, alcohol, smoking), time, time × urbanicity, time × baseline memory centered, and time × covariates (age centered, sex, marital status, heart problems, diabetes, depressive symptoms, alcohol, smoking).

^a^The within-person variance is the overall residual variance in memory that is not explained by the model. The initial status variance component is the variance of individuals’ intercepts about the intercept of the average person. Likewise, the rate of change variance component is the variance of individual slopes about the slope of the average person.

*p < .05. **p < .01. ***p < .001.

We also explored the potential interactions between socioeconomic markers (education, household wealth) and urbanicity, but these were nonsignificant in both cohorts.

The pattern of results was maintained when all SES markers were mutually adjusted, except for the role of urbanicity on memory slope in CHARLS, which was further explained by socioeconomic makers (see Supplementary Table S5). Sex-stratified analyses of education and urbanicity also showed a similar pattern of results to the main analyses, although these associations were slightly stronger among Chinese women than men (see Supplementary Tables S6–S9). The results of the third supplementary analysis matched the baseline memory scores and showed steeper memory decline in CHARLS and stronger socioeconomic inequalities in terms of education and wealth for those with medium and higher baseline memory scores in both countries (see Supplementary Tables S10a–c and S11a–c). In contrast, living in rural China had a significant impact on memory decline in both subset samples of participants with lower and higher scores (see Supplementary Tables S12a–c). Finally, the detailed examination of all education levels within each country showed a significant dose–response in baseline memory performance with every increase in education levels in both ELSA and CHARLS, while the effect on memory decline was only observed in CHARLS (see Supplementary Table S13).

## Discussion

This cross-cohort investigation examined various socioeconomic markers and contextual differentials in relation to memory and memory decline over an almost a decade follow-up period within two nationally representative samples of the English and Chinese population, highlighting significant differences in terms of education, wealth, and urbanicity. This comparative examination suggests that the average baseline memory scores were generally lower in China compared with England when controlling for individual markers of socioeconomic position such as education or wealth and a wide range of covariates, including sociodemographic characteristics, health conditions, and lifestyle behaviors, while the rate of memory decline was much steeper for the Chinese counterparts compared to English participants when accounting for education.

The overall findings illustrate substantial advantages conferred by the individual socioeconomic characteristics such as education and wealth in terms of baseline memory in both English and Chinese participants and a slower rate of memory decline in China but not in England. Such differences are particularly interesting because several studies (Christensen et al., 2001; Karlamangla et al., 2009; Piccinin et al., 2013; Ritchie et al., 2016; Tucker-Drob et al., 2009; Van Dijk et al., 2008; Wilson et al., 2009) and systematic reviews (Seblova et al., 2020) have concluded that there is little to no association between education and cognitive decline over multiple measures of cognition in Western countries, similar to what we found in the English population in the present study. It appears that China is different in this respect, although our findings need further replications.

The specific country differences captured here could be explained by several factors. One is that English participants had overall higher baseline memory scores and declined less over time, while the Chinese respondents started with significantly lower scores and dropped a bit faster. Second, the access to education and pattern of lifestyle behaviors influencing overall health and cognitive performance might be different between England and China. Furthermore, the difference in baseline memory scores could be related to the overall lower level of literacy in China (up to 70%–80% of the population; Wu et al., 2017). Further research unraveling these processes and differences would require more detailed data on childhood cognition, parental social class, interactions with lifestyle behaviors, social mobility, social networks, and country-specific regulations on educational access across generations.

Although individual- and contextual-level characteristics appear to differ in how strong and complex the associations were with memory decline over time, the overall message from the independent models across these two countries was that participants living in rural China were particularly disadvantaged in both baseline memory and the rates of memory decline over time compared with those living in urban China. By contrast, people living in rural areas of England had better memory performance than those living in towns or cities. This is not entirely surprising for China, considering that their rural population consists primarily of farmers and people working in agriculture with less access to education and cultural engagement. Current estimates suggest that 35% of Chinese people work in agriculture, compared with 1.5% in the United Kingdom. Notably, the rural advantage in England was maintained even after controlling for education and individual level of wealth in our mutually adjusted analyses of all SES markers, so selection factors are unlikely to explain these differences. One possibility is that older people living in rural areas of England may benefit from greater access to outdoor green spaces (Besser, 2021).

Our results revealed an interesting dynamic socioeconomic pattern of change in memory among English and Chinese participants with a tendency for marked discrepancy influences in the urban and rural areas, but not at individual-level characteristics (education and wealth) in England, whereas the associations were more uniform in China at both individual- and group-level SES characteristics. Overall results indicate that both English and Chinese middle-aged and older participants benefitted from better memory scores at baseline with higher levels of education and wealth. Among Chinese individuals with relatively no education, higher levels of education were associated with slower linear decline coupled, and a similar pattern was also observed for those with the highest level of wealth. However, the most substantial effects were observed for individuals living in rural China, who experienced the most substantial memory decline over time compared to those living in an urban area. It is important to acknowledge the considerable income disparity between China’s rural and urban residents driven by the residency (hukou) and health care systems. This could be related to the geographic disparities in health status, differences in government expenditure between urban and rural areas, as well as population lifestyle and personal behaviors that could contribute to chronic illness and mental decline (Liu et al., 2015). Furthermore, our sex-stratified investigations confirmed educational and regional discrepancies are affecting women slightly more than men, especially in rural China. These findings are supported by the results of the recent meta-analysis showing an increased risk of MCI in Chinese women, particularly those with low education and rural residents (Lu et al., 2021).

A growing body of evidence has suggested that an individual sustained cultural and educational engagement across life could help build cognitive reserve and mental capital, sculpting an individual’s brain architecture and offering increased neurogenesis (Milgram et al., 2006) and resilience (Negash et al., 2012) when facing the neurodegenerative processes occurring with aging. SES is an important determinant of health and a feature of personal identity with significant influences on cognitive performance, well-being, and social connections for individuals around the world (Braveman & Gottlieb, 2014). The type of residence, lifestyle, and geographical location are reliant on the individual markers of SES (44), and these aspects are usually strongly intertwined. Moreover, contextual-level characteristics such as urbanicity could also influence access to education, income, and wealth, with additional impacts on mental and physical health.

Neuroimaging studies show a strong link between brain structure and level of educational attainment in adulthood, with increases in white matter integrity and fractional anisotropy as well as a decrease in mean diffusivity (Gianaros et al., 2013). Relevant evidence has also been provided by studies investigating the link between community-level socioeconomic factors and brain structure, suggesting reduced cortical volume with a higher level of community deprivation and disadvantage (Krishnadas et al., 2013). The links between socioeconomic disadvantage, brain, and cognitive performance could also be explained by several biological mechanisms such as neuroimmune, neuroendocrine, and cardiometabolic mediating pathways.

### Strengths and Limitations

Our conclusions should be interpreted in light of a number of limitations that need to be acknowledged. We relied on self-report measures of education and household wealth, although we benefitted from objective records for memory and urbanicity. Furthermore, although we controlled for several important covariates known to be associated with both socioeconomic circumstances and memory, the possibility of some biases arising from attrition, survival effects, or uncontrolled confounding may not have been accounted for. Even though the mixed model analyses use maximum likelihood estimation to account for attrition over the 8-year follow-up period, the population sample may be initially selected on critical variables. Those with more education, better financial circumstances, and better memory were more likely to accept the initial invitation to participate in the study. Another limitation of this study is the relatively low representation of mixed ethnicity within ELSA. Furthermore, both ELSA and CHARLS did not cover participants living in institutional settings, so the current results can only be generalized to older people living in the community in England and China, respectively. However, this comparative cross-country investigation was matched in terms of data harmonization for all measures and the follow-up period across the two studies between 2011 and 2019, although independent observation of ELSA would have benefited from a more extended follow-up study period (2002–2019).

Strengths of our study include the coordinated approach analysis of the same cognitive measure and choice of covariates in each of these two nationally representative samples of English and Chinese middle-aged and older populations. Another strength of this study is the use of several measurement occasions to objectively measure memory change (five time points in ELSA and four in CHARLS) and the rich exploration of various baseline socioeconomic markers within each of these cohorts. To the best of our knowledge, this is the first investigation to explore comparatively the role of multiple socioeconomic and contextual markers on cognitive decline in England and China. To untangle their underlying influence on the level of baseline memory and rate of decline, we employed linear mixed models over five measurement occasions in England spanning over 8 years and four measurement occasions in China spanning across a 7-year follow-up period. Mixed linear methods represent a robust measure of examining change in repeated measures of cognition over time when investigating association mapping in the presence of geographic population structure and/or another cryptic relatedness. A particular advantage is that mixed linear models prevent false-positive associations due to these types of systems and provide an increased power specific to the sample structure (Yu et al., 2006). To avoid practice effects, the memory assessments employed by these studies used alternative lists of words at each wave and have undergone monthly quality control to check for inter- and intrarater reliability. Furthermore, the 2- to 3-year gap between memory assessments in these cohorts’ design indicates a reduced possibility of retest effects, which is common in longitudinal studies of cognitive aging (Salthouse, 2014).

## Conclusion

In English and Chinese nationally representative populations, we found strong socioeconomic and contextual differentials as indicated by the level of education, household wealth, and urbanicity affecting memory performance in both countries and the rate of decline, particularly in China. Educational and regional public health policies within each country play a key role in explaining observed differences in health gaps, and substantial efforts should be considered to reduce these inequalities worldwide.

## Supplementary Material

gbac163_suppl_Supplementary_Material

## Author Note

1. Anchoring vignettes are descriptions of hypothetical cases characterized by the most salient objective features of a person or a situation.

## ELSA Data Sharing

The ELSA data are freely available and can be accessed through the UK Data Service (SN 8688 and 5050): https://discover.ukdataservice.ac.uk or via the Gateway to Global Aging data: https://g2aging.org/.

## CHARLS Data Sharing

The CHARLS data are freely available and can be accessed via the CHARLS study data website: http://charls.pku.edu.cn/index/en.html or via the Gateway to Global Aging data: https://g2aging.org/.
