# Supplementary material for: Socioeconomic and Contextual Differentials in Memory Decline: A Cross-Country Investigation Between England and China
Source: J Gerontol B Psychol Sci Soc Sci. 2023 Jan 10;78(3):544–55. doi: 10.1093/geronb/gbac163 (PMC9985334; doi:10.1093/geronb/gbac163)
Supplement: gbac163_suppl_Supplementary_Material [file gbac163_suppl_supplementary_material.docx]

**Supplementary material**

### Socioeconomic indicators-Cross-cohort harmonization process of data derivation

Educational attainment was originally classified into ten categories in CHARLS and seven categories in ELSA. For harmonization purposes, a similar classification was sought within each cohort, and therefore the original cohort classifications were further regrouped into four common categories: no qualification; low level (0-6 years); medium level (7-11 years), and high level of education (12+ years). The breakdown of the participants captured into the final analytical sample of each cohort and the process of harmonization across the two cohorts is further presented in Table S1 below.

Urbanicity was originally classified in ELSA into four categories: Urban, Town & Fringe, Village, and Hamlet and isolated dwelling. However, in CHARLS, the urbanicity was only coded into urban and rural; therefore, a regrouping into urban versus rural was also considered in ELSA, as presented in Table S2.


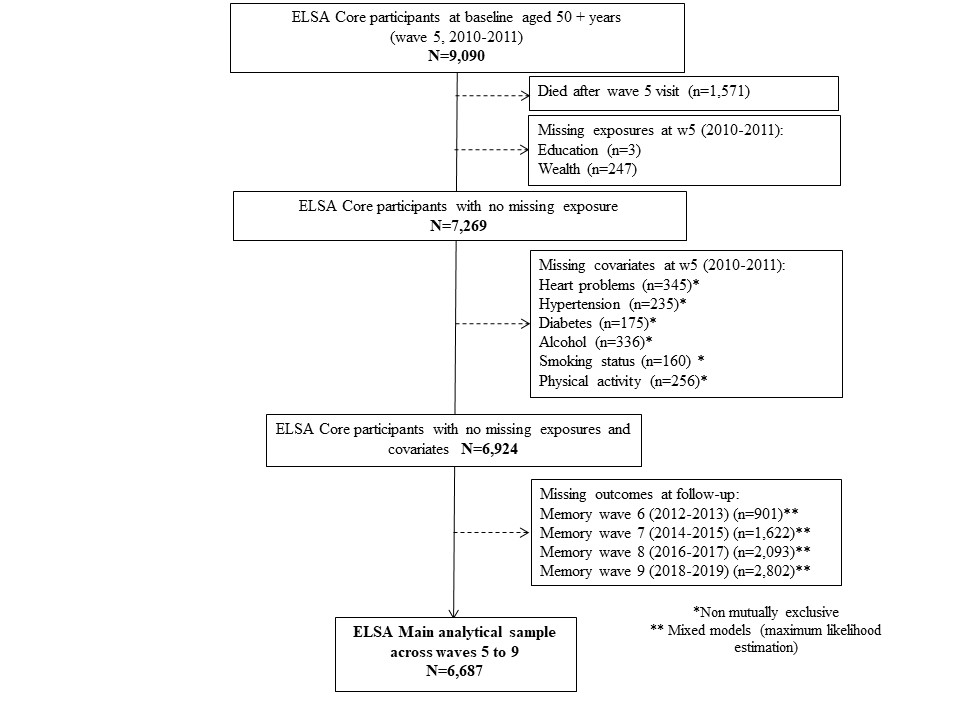


Figure S1. Flow chart of participants selection in ELSA


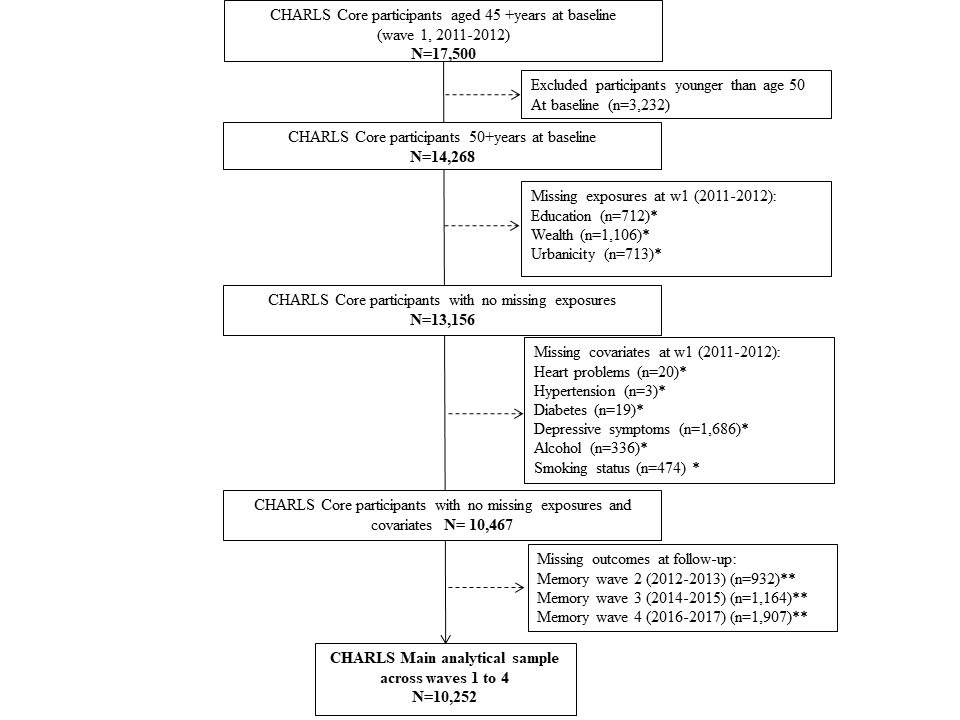


Figure S2. Flow chart of participants selection in CHARLS

Table S1. Harmonization procedure achieved for education classification across ELSA and CHARLS

| ELSA (n=6,687) | | n | | | Harmonized | | Years* |
| --- | --- | --- | --- | --- | --- | --- | --- |
| 1. | No qualification | | 1,686 | 1 | | No education | 0 |
| 2. | Foreign/ other | | 543 | 2 | | Low level | 1-6 |
| 3. | NVQ1/CSE or another grade equivalent | | 289 | 2 | | Low level | 1-6 |
| 4. | NVQ2/GCE O Level equivalent | | 1,313 | 2 | | Low level | 1-6 |
| 5. | NVQ3/GCE A Level equivalent | | 550 | 3 | | Medium level | 7-11 |
| 6. | Higher ed below degree | | 1,023 | 3 | | Medium level | 7-11 |
| 7. | NVQ4/NVQ5/Degree or equivalent | | 1,283 | 4 | | Degree | 12+ |
| CHARLS (n=10,252) | | |  | Harmonized | | | Years* |
| 1. | No formal education, illiterate | | 3,207 | 1 | | No education | 0 |
| 2. | Did not finish primary school | | 1,949 | 2 | | Low level | 1-6 |
| 3. | Sishu | | 55 | 2 | | Low level | 1-6 |
| 4. | Elementary school | | 2,217 | 2 | | Low level | 1-6 |
| 5. | Middle school | | 1,689 | 3 | | Medium level | 7-11 |
| 6. | High school | | 659 | 3 | | Medium level | 7-11 |
| 7. | Vocational school | | 268 | 4 | | Degree | 12+ |
| 8. | Two/Three Year College | | 131 | 4 | | Degree | 12+ |
| 9. | Four Year College/Bachelor’s degree | | 72 | 4 | | Degree | 12+ |
| 10. | Postgraduate, Master’s degree | | 5 | 4 | | Degree | 11+ |

*Approximate number of years for each education category

Table S2. Harmonization procedure achieved for urbanicity classification across ELSA and CHARLS

| ELSA (n=6,687) | | n | | Harmonized | | |
| --- | --- | --- | --- | --- | --- | --- |
| 1. | Urban | 4,837 | | 1 | Urban | |
| 2. | Town & Fringe | 864 | | 2 | Rural | |
| 3. | Village | 714 | | 2 | Rural | |
| 4. | Hamlet and isolated dwelling | 272 | | 2 | Rural | |
| CHARLS (n=10,252) | | |  | Harmonized | | |
| 1. | Urban | 2,371 | | 1 | | Urban |
| 2. | Rural | 7,881 | | 2 | | Rural |

Table S3. Participants’ number and memory scores across waves in ELSA

| **ELSA** | | | | | |  |
| --- | --- | --- | --- | --- | --- | --- |
| **Variables** | **Wave 5 (2010-2011)** | **Wave 6**  **(2012-2013)** | **Wave 7**  **(2014-2015)** | **Wave 8 (2016-2017)** | **Wave 9**  **(2018-2019)** | |
| Subjects, n | 6,687 | 6,023 | 5,276 | 4,818 | 4,121 | |
| Memory, mean (SD) | 10.5 (3.55) | 10.6 (3.6) | 10.3 (3.7) | 10.0 (4.11) | 10.2 (3.7) | |

Table S4. Participants’ number and memory scores across waves in CHARLS

| **CHARLS** | | | | |
| --- | --- | --- | --- | --- |
| **Variables** | **Wave 1**  **(2011-2012)** | **Wave 2**  **(2013)** | **Wave 3**  **(2015)** | **Wave 4**  **(2018)** |
| Subjects, n | 10,252 | 8,444 | 8,212 | 7,469 |
| Memory, mean (SD) | 6.86 (3.34) | 6.70 (3.50) | 5.97 (3.65) | 5.31 (4.50) |

Table S5. Linear mixed models of all the socioeconomic markers (mutually adjusted) predicting memory over time in ELSA and CHARLS

|  | **ELSA (n=6,687)** | **CHARLS (n=10,252)** |
| --- | --- | --- |
|  | **Memory** | **Memory** |
| **Initial status** | **Coefficient (95% CI)** | **Coefficient (95% CI)** |
| Intercept | 7.64 (7.26 to 8.02) *** | 6.87 (6.42 to 7.32) *** |
| Education |  |  |
| No qualification | 1 (ref) | 1 (ref) |
| Low level | 0.92 (0.73 to 1.11) *** | 0.99 (0.86 to 1.14) *** |
| Medium level | 1.40 (1.19 to 1.61) *** | 1.91 (1.74 to 2.09) *** |
| High level | 2.01 (1.77 to 2.25) *** | 3.14 (2.84 to 3.44) *** |
| Wealth |  |  |
| Lowest quintile | 1 (ref) | 1 (ref) |
| 2^nd^ lowest | 0.21 (-0.02 to 0.45) | -0.02 (-0.19 to 0.15) |
| 3^rd^ | 0.56 (0.32 to 0.80) *** | -0.006 (-0.18 to 0.17) |
| 4^th^ highest | 0.80 (0.55 to 1.05) *** | -0.03 (-0.21 to 0.15) |
| 5^th^ highest | 0.99 (0.73 to 1.25) *** | 0.28 (0.09 to 0.47) ** |
| Urbanicity |  |  |
| Urban | 1 (ref) | 1 (ref) |
| Rural | 0.24 (0.09 to 0.40) ** | -0.64 (-0.78 to -0.49) *** |
| **Rate of linear change** | -0.22 (-0.29 to -0.15) *** | -0.39 (-0.50 to -0.27) *** |
| Education |  |  |
| No qualification | 1 (ref) | 1 (ref) |
| Low level | 0.03 (-0.004 to 0.06) | 0.20 (0.17 to 0.24) *** |
| Medium level | 0.02 (-0.01 to 0.06) | 0.33 (0.28 to 0.37) *** |
| High level | 0.03 (-0.01 to 0.07) | 0.27 (0.18 to 0.35) *** |
| Wealth |  |  |
| Lowest quintile | 1 (ref) | 1 (ref) |
| 2^nd^ lowest | 0.04 (-0.005 to 0.07) | 0.03 (-0.01 to 0.07) |
| 3^rd^ | -0.009 (-0.05 to 0.03) | 0.03 (-0.01 to 0.06) |
| 4^th^ highest | 0.02 (-0.03 to 0.06) | 0.04 (-0.01 to 0.07) |
| 5^th^ highest | 0.01 (-0.02 to 0.07) | 0.06 (0.02 to 0.11)** |
| Urbanicity |  |  |
| Urban | 1 (ref) | 1 (ref) |
| Rural | -0.01 (-0.04 to 0.01) | -0.04 (-0.08 to 0.003) |
| **Variance ^a^** |  |  |
| Within-person | 4.97 (4.80 to 5.15) | 6.81 (6.66 to 6.97) |
| In initial status | 4.54 (4.21 to 4.85) | 2.87 (2.63 to 3.12) |
| In rate of change | 0.04 (0.02 to 0.06) | 0.16 (0.14 to 0.18) |
| **Goodness of fit** |  |  |
| Deviance (-2LL ^a^) | -63,622.82 | -85,277.72 |
| Wald chi2(29) | 4,094.55 | 9,335.14 |
| P-value | ≤0.001 | ≤0.001 |

CI, Confidence Intervals; LL, Log-likelihood; AIC, Akaike’s Information Criterion; BIC, Bayesian Information Criterion. The models include memory, SES markers or urbanicity, covariates (age centred, sex, marital status, heart problems, diabetes, depressive symptoms, alcohol, smoking), time, time × SES markers or urbanicity, time x baseline memory centred, and time ×covariates (age centred, sex, marital status, heart problems, diabetes, depressive symptoms, alcohol, smoking).

^a^ The within-person variance is the overall residual variance in memory that is not explained by the model. The initial status variance component is the variance of individuals’ intercepts about the intercept of the average person. Likewise, the rate of change variance component is the variance of individual slopes about the slope of the average person.

*p<0.05, **p<0.01, ***p<0.001

Table S6. Linear mixed models of education predicting memory over time in ELSA and CHARLS in men

|  | **ELSA (n=3,047)** | **CHARLS (n=4,824)** |
| --- | --- | --- |
|  | **Memory** | **Memory** |
| **Initial status** | **Coefficient (95% CI)** | **Coefficient (95% CI)** |
| Intercept | 9.49 (9.22 to 9.77) *** | 6.05 (5.80 to 6.30) |
| Education |  |  |
| No qualification | 1 (ref) | 1 (ref) |
| Low level | 0.79 (0.49 to 1.10) *** | 1.05 (0.81 to 1.29)*** |
| Medium level | 1.46 (1.17 to 1.76) *** | 2.05 (1.78 to 2.32) *** |
| High level | 2.25 (1.93 to 2.58) *** | 3.29 (2.91 to 3.66) *** |
| **Rate of linear change** | -0.20 (-0.25 to -0.15) *** | -0.36 (-0.43 to -0.30) *** |
| Education |  |  |
| No qualification | 1 (ref) | 1 (ref) |
| Low level | 0.03 (-0.03 to 0.08) | 0.13 (0.07 to 0.19) *** |
| Medium level | 0.02 (-0.03 to 0.08) | 0.23 (0.16 to 0.30) *** |
| High level | 0.03 (-0.03 t 0.09) | 0.19 (0.09 to 0.29) *** |
| **Variance ^a^** |  |  |
| Within-person | 4.66 (4.41 to 4.91) | 6.64 (6.43 to 6.87) |
| In initial status | 4.53 (4.13 to 4.98) | 2.72 (2.39 to 3.09) |
| In rate of change | 0.04 (0.03 to 0.08) | 0.17 (0.14 to 0.20) |
| **Goodness of fit** |  |  |
| Deviance (-2LL ^a^) | -29,927.20 | 39,944.29 |
| Wald chi2(29) | 1,695.69 | 3,201.25 |
| P-value | ≤0.001 | ≤0.001 |

CI, Confidence Intervals; LL, Log-likelihood; AIC, Akaike’s Information Criterion; BIC, Bayesian Information Criterion. The models include memory, education, covariates (age centred, marital status, heart problems, diabetes, depressive symptoms, alcohol, smoking), time, time ×education, time x baseline memory centred, and time ×covariates (age centred, marital status, heart problems, diabetes, depressive symptoms, alcohol, smoking).

^a^ The within-person variance is the overall residual variance in memory that is not explained by the model. The initial status variance component is the variance of individuals’ intercepts about the intercept of the average person. Likewise, the rate of change variance component is the variance of individual slopes about the slope of the average person.

*p<0.05, **p<0.01, ***p<0.001

Table S7. Linear mixed models of education predicting memory over time in ELSA and CHARLS in women

|  | **ELSA (n=3,640)** | **CHARLS (n=5,428)** |
| --- | --- | --- |
|  | **Memory** | **Memory** |
| **Initial status** | **Coefficient (95% CI)** | **Coefficient (95% CI)** |
| Intercept | 10.18 (9.96 to 10.39) *** | 6.09 (5.96 to 6.24) *** |
| Education |  |  |
| No qualification | 1 (ref) | 1 (ref) |
| Low level | 1.18 (0.99 to 1.42) *** | 1.06 (0.88 to 1.24) *** |
| Medium level | 1.72 (1.44 to 2.09) *** | 2.31 (2.08 to 2.54) *** |
| High level | 2.40 (2.07 to 2.73) *** | 4.36 (3.86 to 4.87) *** |
| **Rate of linear change** | -0.16 (-0.20 to -0.13) *** | -0.44 (-0.48 to -0.41) *** |
| Education |  |  |
| No qualification | 1 (ref) | 1 (ref) |
| Low level | 0.03 (-0.01 to 0.07) | 0.22 (0.18 to 0.27) *** |
| Medium level | 0.03 (-0.02 to 0.07) | 0.39 (0.33 to 0.45) *** |
| High level | 0.04 (-0.02 to 0.09) | 0.32 (0.19 to 0.45) *** |
| **Variance ^a^** |  |  |
| Within-person | 5.27 (5.04 to 5.52) | 6.96 (6.75 to 7.18) |
| In initial status | 4.70 (4.28 to 5.17) | 3.12 (2.79 to 3.49) |
| In rate of change | 0.03 (0.01 to 0.08) | 0.15 (0.13 to 0.19) |
| **Goodness of fit** |  |  |
| Deviance (-2LL ^a^) | -33,732.14 | -45,391.05 |
| Wald chi2(29) | 2,188.30 | 5,777.07 |
| P-value | ≤0.001 | ≤0.001 |

CI, Confidence Intervals; LL, Log-likelihood; AIC, Akaike’s Information Criterion; BIC, Bayesian Information Criterion. The models include memory, education, covariates (age centred, marital status, heart problems, diabetes, depressive symptoms, alcohol, smoking), time, time ×education, time x baseline memory centred, and time ×covariates (age centred, marital status, heart problems, diabetes, depressive symptoms, alcohol, smoking).

^a^ The within-person variance is the overall residual variance in memory that is not explained by the model. The initial status variance component is the variance of individuals’ intercepts about the intercept of the average person. Likewise, the rate of change variance component is the variance of individual slopes about the slope of the average person.

*p<0.05, **p<0.01, ***p<0.001

Table S8. Linear mixed models of urbanicity predicting memory over time in ELSA and CHARLS in men

|  | **ELSA (n=3,047)** | **CHARLS (n=4,824)** |
| --- | --- | --- |
|  | **Memory** | **Memory** |
| **Initial status** | **Coefficient (95% CI)** | **Coefficient (95% CI)** |
| Intercept | 10.36 (10.03 to 10.70) *** | 9.82 (9.44 to 10.19) *** |
| Urbanicity |  |  |
| Urban | 1 (ref) | 1 (ref) |
| Rural | 0.29 (0.06 to 0.52) * | -1.31 (-1.51 to -1.14) *** |
| **Rate of linear change** | -0.14 (-0.22 to -0.06) *** | -0.14 (-0.25 to -0.04) |
| Urbanicity |  |  |
| Urban | 1 (ref) | 1 (ref) |
| Rural | -0.008 (-0.05 to 0.03) | -0.04 (-0.09 to 0.01) *** |
| **Variance ^a^** |  |  |
| Within-person | 4.65 (4.41 to 4.91) | 6.64 (6.43 to 6.87) |
| In initial status | 5.07 (4.64 to 5.54) | 3.08 (2.75 to 3.46) |
| In rate of change | 0.05 (0.03 to 0.08) | 0.20 (0.16 to 0.24) |
| **Goodness of fit** |  |  |
| Deviance (-2LL ^a^) | -30,054.51 | -40,240.64 |
| Wald chi2(47) | 1,415.41 | 2,385.29 |
| P-value | ≤0.001 | ≤0.001 |

CI, Confidence Intervals; LL, Log-likelihood; AIC, Akaike’s Information Criterion; BIC, Bayesian Information Criterion. The models include memory, urbanicity, covariates (age centred, marital status, heart problems, diabetes, depressive symptoms, alcohol, smoking), time, time × urbanicity, time x baseline memory centred, and time ×covariates (age centred, marital status, heart problems, diabetes, depressive symptoms, alcohol, smoking).

^a^ The within-person variance is the overall residual variance in memory that is not explained by the model. The initial status variance component is the variance of individuals’ intercepts about the intercept of the average person. Likewise, the rate of change variance component is the variance of individual slopes about the slope of the average person.

*p<0.05, **p<0.01, ***p<0.001

Table S9. Linear mixed models of urbanicity predicting memory over time in ELSA and CHARLS in women

|  | **ELSA (n=3,640)** | **CHARLS (n=5,428)** |
| --- | --- | --- |
|  | **Memory** | **Memory** |
| **Initial status** | **Coefficient (95% CI)** | **Coefficient (95% CI)** |
| Intercept | 10.66 (10.33 to 10.98) *** | 9.64 (9.29 to 9.99) *** |
| Urbanicity |  |  |
| Urban | 1 (ref) | 1 (ref) |
| Rural | 0.52 (0.29 to 0.73) *** | -1.49 (-1.69 to -1.31) *** |
| **Rate of linear change** | -0.17 (-0.25 to -0.09) *** | -0.11 (-0.20 to -0.01) ** |
| Urbanicity |  |  |
| Urban | 1 (ref) | 1 (ref) |
| Rural | -0.01 (-0.05 to 0.02) | -0.10 (-0.16 to -0.05) *** |
| **Variance ^a^** |  |  |
| Within-person | 5.27 (5.04 to 5.52) | 6.97 (6.75 to 7.18) |
| In initial status | 5.23 (4.79 to 5.72) | 3.66 (3.31 to 4.05) |
| In rate of change | 0.04 (0.02 to 0.08) | 0.22 (0.19 to 0.26) |
| **Goodness of fit** |  |  |
| Deviance (-2LL ^a^) | -33,881.58 | -46,045.95 |
| Wald chi2(47) | 1,809.73 | 3,702.25 |
| P-value | ≤0.001 | ≤0.001 |

CI, Confidence Intervals; LL, Log-likelihood; AIC, Akaike’s Information Criterion; BIC, Bayesian Information Criterion. The models include memory, urbanicity, covariates (age centred, marital status, heart problems, diabetes, depressive symptoms, alcohol, smoking), time, time × urbanicity, time x baseline memory centred, and time ×covariates (age centred, marital status, heart problems, diabetes, depressive symptoms, alcohol, smoking).

^a^ The within-person variance is the overall residual variance in memory that is not explained by the model. The initial status variance component is the variance of individuals’ intercepts about the intercept of the average person. Likewise, the rate of change variance component is the variance of individual slopes about the slope of the average person.

*p<0.05, **p<0.01, ***p<0.001

Table S10a. Linear mixed models of education predicting memory over time in ELSA and CHARLS – matched analyses for low baseline performance (scores ≤9)

|  | **ELSA (n=2,377)** | **CHARLS (n=8,138)** |
| --- | --- | --- |
|  | **Memory** | **Memory** |
| **Initial status** | **Coefficient (95% CI)** | **Coefficient (95% CI)** |
| Intercept | 7.28 (6.92 to 7.64) *** | 5.34 (5.07 to 5.62) *** |
| Education |  |  |
| No qualification | 1 (ref) | 1 (ref) |
| Low level | 0.45 (0.24 to 0.67) *** | 0.67 (0.54 to 0.79) *** |
| Medium level | 0.76 (0.50 to 1.02) *** | 1.19 (1.03 to 1.35) *** |
| High level | 0.64 (0.30 to 0.92) *** | 2.12 (1.79 to 2.43) *** |
| **Rate of linear change** | -0.15 (-0.24 to -0.05) *** | -0.37 (-0.45 to -0.28) *** |
| Education |  |  |
| No qualification | 1 (ref) | 1 (ref) |
| Low level | 0.03 (-0.02 to 0.08) | 0.26 (0.22 to 0.24) *** |
| Medium level | 0.04 (-0.03 to 0.10) | 0.48 (0.44 to 0.56) *** |
| High level | 0.10 (0.03 to 0.18)* | 0.44 (0.34 to 0.53) *** |
| **Variance ^a^** |  |  |
| Within-person | 5.10 (4.83 to 5.41) | 6.41 (6.25 to 6.57) |
| In initial status | 1.66 (1.35 to 2.04) | 0.63 (0.46 to 0.86) |
| In rate of change | 0.04 (0.04 to 0.06) | 0.09 (0.08 to 0.11) |
| **Goodness of fit** |  |  |
| Deviance (-2LL ^a^) | -20,672.09 | -65,289.87 |
| Wald chi2(29) | 729.65 | 5,072.22 |
| P-value | ≤0.001 | ≤0.001 |

CI, Confidence Intervals; LL, Log-likelihood; AIC, Akaike’s Information Criterion; BIC, Bayesian Information Criterion. The models include memory, education, covariates (age centred, marital status, heart problems, diabetes, depressive symptoms, alcohol, smoking), time, time ×education, time x baseline memory centred, and time ×covariates (age centred, marital status, heart problems, diabetes, depressive symptoms, alcohol, smoking).

^a^ The within-person variance is the overall residual variance in memory that is not explained by the model. The initial status variance component is the variance of individuals’ intercepts about the intercept of the average person. Likewise, the rate of change variance component is the variance of individual slopes about the slope of the average person.

*p<0.05, **p<0.01, ***p<0.001

Table S10b. Linear mixed models of education predicting memory over time in ELSA and CHARLS – matched analyses for medium baseline performance (scores 10-12)

|  | **ELSA (n=2,370)** | **CHARLS (n=1,628)** |
| --- | --- | --- |
|  | **Memory** | **Memory** |
| **Initial status** | **Coefficient (95% CI)** | **Coefficient (95% CI)** |
| Intercept | 10.52 (10.28 to 10.75) *** | 9.80 (9.25 to 10.35) *** |
| Education |  |  |
| No qualification | 1 (ref) | 1 (ref) |
| Low level | 0.12 (-0.03 to 0.27) | 0.05 (-0.26 to 0.37) |
| Medium level | 0.28 (0.10 to 0.44) *** | 0.49 (0.17 to 0.83) ** |
| High level | 0.51 (0.34 to 0.69) *** | 0.75 (0.32 to 1.18) *** |
| **Rate of linear change** | -0.40 (-0.48 to -0.32) *** | -1.26 (-1.49 to -1.02) *** |
| Education |  |  |
| No qualification | 1 (ref) | 1 (ref) |
| Low level | 0.11 (0.06 to 0.17) *** | 0.49 (0.39 to 0.60) *** |
| Medium level | 0.17 (0.11 to 0.23) *** | 0.74 (0.63 to 0.85) *** |
| High level | 0.19 (0.13 to 0.25) *** | 0.95 (0.80 to 1.09) *** |
| **Variance ^a^** |  |  |
| Within-person | 3.82 (3.63 to 4.02) | 5.49 (5.25 to 5.73) |
| In initial status | 0.16 (0.11 to 0.22) | 0.03 (0.009 to 0.11) |
| In rate of change | 0.08 (0.07 to 0.09) | 0.17 (0.14 to 0.20) |
| **Goodness of fit** |  |  |
| Deviance (-2LL ^a^) | -21,282.28 | -13,201.33 |
| Wald chi2(29) | 795.59 | 1,375.27 |
| P-value | ≤0.001 | ≤0.001 |

CI, Confidence Intervals; LL, Log-likelihood; AIC, Akaike’s Information Criterion; BIC, Bayesian Information Criterion. The models include memory, education, covariates (age centred, marital status, heart problems, diabetes, depressive symptoms, alcohol, smoking), time, time ×education, time x baseline memory centred, and time ×covariates (age centred, marital status, heart problems, diabetes, depressive symptoms, alcohol, smoking).

^a^ The within-person variance is the overall residual variance in memory that is not explained by the model. The initial status variance component is the variance of individuals’ intercepts about the intercept of the average person. Likewise, the rate of change variance component is the variance of individual slopes about the slope of the average person.

*p<0.05, **p<0.01, ***p<0.001

Table S10c. Linear mixed models of education predicting memory over time in ELSA and CHARLS – matched analyses for medium baseline performance (scores 13+)

|  | **ELSA (n=5,649)** | **CHARLS (n=1,607)** |
| --- | --- | --- |
|  | **Memory** | **Memory** |
| **Initial status** | **Coefficient (95% CI)** | **Coefficient (95% CI)** |
| Intercept | 13.50 (13.05 to 13.96) *** | 11.74 (10.49 to 13.06) *** |
| Education |  |  |
| No qualification | 1 (ref) | 1 (ref) |
| Low level | 0.15 (-0.15 to 0.46) | 0.18 (-0.64 to 1.09) |
| Medium level | 0.42 (0.08 to 0.75) * | 0.43 (-0.39 to 1.24) |
| High level | 0.74 (0.40 to 1.08) *** | 1.43 (0.49 to 2.39) ** |
| **Rate of linear change** | -0.77 (-0.91 to -0.63) *** | -1.75 (-2.23 to -1.27) *** |
| Education |  |  |
| No qualification | 1 (ref) | 1 (ref) |
| Low level | 0.16 (0.05 to 0.27) *** | 0.55 (0.30 to 0.79) *** |
| Medium level | 0.17 (0.06 to 0.28) *** | 0.67 (0.43 to 0.91) *** |
| High level | 0.22 (0.11 to 0.32) *** | 0.84 (0.54 to 1.13) *** |
| **Variance ^a^** |  |  |
| Within-person | 4.83 (4.48 to 5.22) | 8.26 (7.61 to 8.97) |
| In initial status | 0.31 (0.18 to 0.53) | 0.001 (0.001 to 0.006) |
| In rate of change | 0.06 (0.05 to 0.08) | 0.17 (0.12 to 0.22) |
| **Goodness of fit** |  |  |
| Deviance (-2LL ^a^) | -12375.77 | -41,47.77 |
| Wald chi2(29) | 888.55 | 588.20 |
| P-value | ≤0.001 | ≤0.001 |

CI, Confidence Intervals; LL, Log-likelihood; AIC, Akaike’s Information Criterion; BIC, Bayesian Information Criterion. The models include memory, education, covariates (age centred, marital status, heart problems, diabetes, depressive symptoms, alcohol, smoking), time, time ×education, time x baseline memory centred, and time ×covariates (age centred, marital status, heart problems, diabetes, depressive symptoms, alcohol, smoking).

^a^ The within-person variance is the overall residual variance in memory that is not explained by the model. The initial status variance component is the variance of individuals’ intercepts about the intercept of the average person. Likewise, the rate of change variance component is the variance of individual slopes about the slope of the average person.

*p<0.05, **p<0.01, ***p<0.001

Table S11a. Linear mixed models of household wealth predicting memory over time in ELSA and CHARLS – matched analyses for low baseline performance (scores ≤9)

|  | **ELSA (n=2,377)** | **CHARLS (n=8,138)** |
| --- | --- | --- |
|  | **Memory** | **Memory** |
| **Initial status** | **Coefficient (95% CI)** | **Coefficient (95% CI)** |
| Intercept | 7.37 (6.98 to 7.77) *** | 6.36 (6.10 to 6.62) *** |
| Wealth |  |  |
| Lowest quintile | 1 (ref) | 1 (ref) |
| 2^nd^ lowest | 0.23 (-0.05 to 0.51) | 0.06 (-0.10 to 0.22) |
| 3^rd^ | 0.34 (0.05 to 0.63) * | 0.05 (-0.12 to 0.22) |
| 4^th^ highest | 0.68 (0.37 to 0.98) *** | 0.23 (0.06 to 0.39) ** |
| 5^th^ highest | 0.70 (0.36 to 1.05) *** | 0.46 (0.29 to 0.63) *** |
| **Rate of linear change** | -0.13 (-0.24 to -0.03) * | -0.02 (-0.06 to 0.10) |
| Wealth |  |  |
| Lowest quintile | 1 (ref) | 1 (ref) |
| 2^nd^ lowest | 0.03 (-0.04 to 0.09) | 0.02 (-0.03 to 0.07) |
| 3^rd^ | 0.01 (-0.06 to 0.08) | 0.05 (0.004 to 0.10)* |
| 4^th^ highest | 0.05 (-0.02 to 0.12) | 0.08 (0.03 to 0.13)** |
| 5^th^ highest | 0.07 (-0.001 to 0.15) | 0.10 (0.05 to 0.15)*** |
| **Variance ^a^** |  |  |
| Within-person | 5.11 (4.83 to 5.41) | 6.41 (6.25 to 6.57) |
| In initial status | 1.67 (1.36 to 2.07) | 0.81 (0.63 to .04) |
| In rate of change | 0.05 (0.04 to 0.06) | 0.14 (0.12 to 0.16) |
| **Goodness of fit** |  |  |
| Deviance (-2LL ^a^) | -20,680.77 | -66,037.28 |
| Wald chi2(29) | 709.06 | 3,003.83 |
| P-value | ≤0.001 | ≤0.001 |

CI, Confidence Intervals; LL, Log-likelihood; AIC, Akaike’s Information Criterion; BIC, Bayesian Information Criterion. The models include memory, wealth, covariates (age centred, marital status, heart problems, diabetes, depressive symptoms, alcohol, smoking), time, time ×wealth, time x baseline memory centred, and time ×covariates (age centred, marital status, heart problems, diabetes, depressive symptoms, alcohol, smoking).

^a^ The within-person variance is the overall residual variance in memory that is not explained by the model. The initial status variance component is the variance of individuals’ intercepts about the intercept of the average person. Likewise, the rate of change variance component is the variance of individual slopes about the slope of the average person.

*p<0.05, **p<0.01, ***p<0.001

Table S11b. Linear mixed models of household wealth predicting memory over time in ELSA and CHARLS – matched analyses for medium baseline performance (scores 10-12)

|  | **ELSA (n=2,370)** | **CHARLS (n=2,519)#** |
| --- | --- | --- |
|  | **Memory** | **Memory** |
| **Initial status** | **Coefficient (95% CI)** | **Coefficient (95% CI)** |
| Intercept | 10.50 (10.25 to 10.75) *** | 9.84 (9.44 to 10.25) *** |
| Wealth |  |  |
| Lowest quintile | 1 (ref) | 1 (ref) |
| 2^nd^ lowest | 0.15 (-0.05 to 0.35) | -0.08 (-0.36 to 0.20) |
| 3^rd^ | 0.38 (0.18 to 0.58) *** | -0.10 (-0.38 to 0.17) |
| 4^th^ highest | 0.25 (0.05 to 0.45) | -0.02 (-0.28 to 0.25) |
| 5^th^ highest | 0.53 (0.33 to 0.73) *** | 0.22 (-0.04 to 0.47) |
| **Rate of linear change** | -0.32 (-0.40 to -0.24) * | -0.55 (-0.71 to -0.37) *** |
| Wealth |  |  |
| Lowest quintile | 1 (ref) | 1 (ref) |
| 2^nd^ lowest | 0.08 (0.02 to 0.15)* | 0.004 (-0.10 to 0.11) |
| 3^rd^ | 0.03 (-0.03 to 0.09) | 0.02 (-0.08 to 0.12) |
| 4^th^ highest | 0.09 (0.02 to 0.15)** | 0.14 (0.05 to 0.24)** |
| 5^th^ highest | 0.13 (0.07 to 0.20)*** | 0.15 (0.06 to 0.24)** |
| **Variance ^a^** |  |  |
| Within-person | 3.82 (3.62 to 4.03) | 5.44 (5.25 to 5.64) |
| In initial status | 0.16 (0.11 to 0.21) | 0.05 (0.02 to 0.12) |
| In rate of change | 0.08 (0.07 to 0.10) | 0.22 (0.20 to 0.25) |
| **Goodness of fit** |  |  |
| Deviance (-2LL ^a^) | -21,303.49 | -20,617.76 |
| Wald chi2(29) | 788.33 | 1,226.12 |
| P-value | ≤0.001 | ≤0.001 |

CI, Confidence Intervals; LL, Log-likelihood; AIC, Akaike’s Information Criterion; BIC, Bayesian Information Criterion. The models include memory, wealth, covariates (age centred, marital status, heart problems, diabetes, depressive symptoms, alcohol, smoking), time, time ×wealth, time x baseline memory centred, and time ×covariates (age centred, marital status, heart problems, diabetes, depressive symptoms, alcohol, smoking).

^a^ The within-person variance is the overall residual variance in memory that is not explained by the model. The initial status variance component is the variance of individuals’ intercepts about the intercept of the average person. Likewise, the rate of change variance component is the variance of individual slopes about the slope of the average person.

*p<0.05, **p<0.01, ***p<0.001

#Scores 9 to 12 for CHARLS & 10 to 12 for ELSA

Table S11c. Linear mixed models of household wealth predicting memory over time in ELSA and CHARLS – matched analyses for medium baseline performance (scores 13+)

|  | **ELSA (n=1,295)** | **CHARLS (n=273)** |
| --- | --- | --- |
|  | **Memory** | **Memory** |
| **Initial status** | **Coefficient (95% CI)** | **Coefficient (95% CI)** |
| Intercept | 13.52 (12.99 to 14.05) *** | 13.38 (11.72 to 15.05) *** |
| Wealth |  |  |
| Lowest quintile | 1 (ref) | 1 (ref) |
| 2^nd^ lowest | 0.31 (-0.09 to 0.70) | -0.92 (-2.17 to 0.32) |
| 3^rd^ | 0.45 (0.06 to 0.84) * | -0.49 (-1.67 to 0.67) |
| 4^th^ highest | 0.59 (0.21 to 0.97)* | -0.05 (-1.19 to 1.10) |
| 5^th^ highest | 0.47 (0.08 to 0.86) * | -0.11 (-1.17 to 0.93) |
| **Rate of linear change** | -0.70 (-0.85 to -0.56) *** | -1.47 (-2.21 to -0.73) *** |
| Wealth |  |  |
| Lowest quintile | 1 (ref) | 1 (ref) |
| 2^nd^ lowest | 0.04 (-0.07 to 0.15) | 0.11 (-0.28 to 0.50) |
| 3^rd^ | 0.06 (-0.05 to 0.18) | -0.09 (-0.46 to 0.27) |
| 4^th^ highest | 0.11 (0.004 to 0.21)* | 0.09 (-0.27 to 0.45) |
| 5^th^ highest | 0.14 (0.03 to 0.25)* | 0.04 (-0.29 to 0.37) |
| **Variance ^a^** |  |  |
| Within-person | 4.85 (4.49 to 5.22) | 9.15 (8.21 to 10.21) |
| In initial status | 0.32 (0.18 to 0.54) | 0.007 (7.9 to 60.23) |
| In rate of change | 0.06 (0.05 to 0.08) | 0.22 (0.14 to 0.34) |
| **Goodness of fit** |  |  |
| Deviance (-2LL ^a^) | -12393.76 | -2,393.97 |
| Wald chi2(29) | 875.45 | 302.39 |
| P-value | ≤0.001 | ≤0.001 |

CI, Confidence Intervals; LL, Log-likelihood; AIC, Akaike’s Information Criterion; BIC, Bayesian Information Criterion. The models include memory, wealth, covariates (age centred, sex, marital status, heart problems, diabetes, depressive symptoms, alcohol, smoking), time, time ×wealth, time x baseline memory centred, and time ×covariates (age centred, sex, marital status, heart problems, diabetes, depressive symptoms, alcohol, smoking).

^a^ The within-person variance is the overall residual variance in memory that is not explained by the model. The initial status variance component is the variance of individuals’ intercepts about the intercept of the average person. Likewise, the rate of change variance component is the variance of individual slopes about the slope of the average person.

*p<0.05, **p<0.01, ***p<0.001

Table S12a. Linear mixed models of urbanicity predicting memory over time in ELSA and CHARLS – matched analyses for low baseline performance (scores ≤9)

|  | **ELSA (n=2,377)** | **CHARLS (n=8,138)** |
| --- | --- | --- |
|  | **Memory** | **Memory** |
| **Initial status** | **Coefficient (95% CI)** | **Coefficient (95% CI)** |
| Intercept | 7.81 (7.41 to 8.21) *** | 7.85 (7.52 to 8.19) *** |
| Urbanicity |  |  |
| Urban | 1 (ref) | 1 (ref) |
| Rural | -0.009 (-0.21 to -0.21) | -0.73 (-0.87 to -0.60) *** |
| **Rate of linear change** | -0.03 (-0.16 to 0.10) | -0.37 (0.26 to 0.47) *** |
| Urbanicity |  |  |
| Urban | 1 (ref) | 1 (ref) |
| Rural | 0.02 (-0.03 to 0.07) | -0.16 (-0.20 to -0.12) *** |
| **Variance ^a^** |  |  |
| Within-person | 5.10 (4.82 to 5.40) | 6.41 (6.25 to 6.58) |
| In initial status | 1.74 (1.42 to 2.14) | 0.75 (0.58 to 0.98) |
| In rate of change | 0.05 (0.04 to 0.06) | 0.14 (0.12 to 0.16) |
| **Goodness of fit** |  |  |
| Deviance (-2LL ^a^) | -20,699.51 | -65,916.68 |
| Wald chi2(29) | 655.12 | 3,363.35 |
| P-value | ≤0.001 | ≤0.001 |

CI, Confidence Intervals; LL, Log-likelihood; AIC, Akaike’s Information Criterion; BIC, Bayesian Information Criterion. The models include memory, urbanicity, covariates (age centred, sex, marital status, heart problems, diabetes, depressive symptoms, alcohol, smoking), time, time × urbanicity, time x baseline memory centred, and time ×covariates (age centred, sex, marital status, heart problems, diabetes, depressive symptoms, alcohol, smoking).

^a^ The within-person variance is the overall residual variance in memory that is not explained by the model. The initial status variance component is the variance of individuals’ intercepts about the intercept of the average person. Likewise, the rate of change variance component is the variance of individual slopes about the slope of the average person.

*p<0.05, **p<0.01, ***p<0.001

Table S12b. Linear mixed models of urbanicity predicting memory over time in ELSA and CHARLS – matched analyses for low baseline performance (scores 10-12)

|  | **ELSA (n=2,370)** | **CHARLS (n=1,628)#** |
| --- | --- | --- |
|  | **Memory** | **Memory** |
| **Initial status** | **Coefficient (95% CI)** | **Coefficient (95% CI)** |
| Intercept | 10.59 (10.34 to 10.83) *** | 10.93 (10.39 to 11.48) *** |
| Urbanicity |  |  |
| Urban | 1 (ref) | 1 (ref) |
| Rural | 0.18 (0.06 to 0.31)* | -0.39 (-0.60 to -0.18) *** |
| **Rate of linear change** | -0.70 (-0.95 to -0.45)*** | 0.07 (-0.18 to 0.33) |
| Urbanicity |  |  |
| Urban | 1 (ref) | 1 (ref) |
| Rural | -0.004 (-0.04 to 0.04) | -0.33 (-0.40 to -0.25) |
| **Variance ^a^** |  |  |
| Within-person | 3.82 (3.63 to 4.03) | 5.49 (5.26 to 5.74) |
| In initial status | 0.17 (0.12 to 0.23) | 0.05 (0.02 to 0.13) |
| In rate of change | 0.08 (0.07 to 0.09) | 0.21 (0.18 to 0.24) |
| **Goodness of fit** |  |  |
| Deviance (-2LL ^a^) | -21,332.96 | -13,316.53 |
| Wald chi2(29) | 704.46 | 1,010.09 |
| P-value | ≤0.001 | ≤0.001 |

CI, Confidence Intervals; LL, Log-likelihood; AIC, Akaike’s Information Criterion; BIC, Bayesian Information Criterion. The models include memory, urbanicity, covariates (age centred, sex, marital status, heart problems, diabetes, depressive symptoms, alcohol, smoking), time, time × urbanicity time x baseline memory centred, and time ×covariates (age centred, sex, marital status, heart problems, diabetes, depressive symptoms, alcohol, smoking).

^a^ The within-person variance is the overall residual variance in memory that is not explained by the model. The initial status variance component is the variance of individuals’ intercepts about the intercept of the average person. Likewise, the rate of change variance component is the variance of individual slopes about the slope of the average person.

*p<0.05, **p<0.01, ***p<0.001

#Scores 9 to 12 for CHARLS & 10 to 12 for ELSA

Table S12c. Linear mixed models of urbanicity predicting memory over time in ELSA and CHARLS – matched analyses for low baseline performance (scores 13+)

|  | **ELSA (n=1,940)** | **CHARLS (n=273)** |
| --- | --- | --- |
|  | **Memory** | **Memory** |
| **Initial status** | **Coefficient (95% CI)** | **Coefficient (95% CI)** |
| Intercept | 13.22 (12.85 to 13.59) *** | 13.82 (12.07 to 15.57) *** |
| Urbanicity |  |  |
| Urban | 1 (ref) | 1 (ref) |
| Rural | -0.17 (-0.001 to 0.34) | -0.45 (-1.09 to 0.17) |
| **Rate of linear change** | -0.94 (-1.14 to -0.74) | -1.11 (-1.86 to -0.36) * |
| Urbanicity |  |  |
| Urban | 1 (ref) | 1 (ref) |
| Rural | 0.001 (-0.04 to 0.04) | -0.24 (-0.44 to -0.04)* |
| **Variance ^a^** |  |  |
| Within-person | 4.74 (4.54 to 5.04) | 9.24 (8.27 to 10.3-) |
| In initial status | 0.51 (0.35 to 0.73) | 0.001 (0.001 to 0.08) |
| In rate of change | 0.06 (0.04 to 0.07) | 0.20 (0.14 to 0.28) |
| **Goodness of fit** |  |  |
| Deviance (-2LL ^a^) | -18,644.75 | -2,389.55 |
| Wald chi2(29) | 1057.01 | 319.45 |
| P-value | ≤0.001 | ≤0.001 |

CI, Confidence Intervals; LL, Log-likelihood; AIC, Akaike’s Information Criterion; BIC, Bayesian Information Criterion. The models include memory, urbanicity, covariates (age centred, sex, marital status, heart problems, diabetes, depressive symptoms, alcohol, smoking), time, time ×urbanicity, time x baseline memory centred, and time ×covariates (age centred, sex, marital status, heart problems, diabetes, depressive symptoms, alcohol, smoking).

^a^ The within-person variance is the overall residual variance in memory that is not explained by the model. The initial status variance component is the variance of individuals’ intercepts about the intercept of the average person. Likewise, the rate of change variance component is the variance of individual slopes about the slope of the average person.

*p<0.05, **p<0.01, ***p<0.001

Table S13. Linear mixed models of education predicting memory over time in ELSA and CHARLS in men

|  | **ELSA (n=6,687)** |  | **CHARLS (n=10,252)** |
| --- | --- | --- | --- |
|  | **Memory** |  | **Memory** |
| **Initial status** | **Coefficient (95% CI)** | **Initial status** | **Coefficient (95% CI)** |
| Intercept | 8.48 (8.20 to 8.76) *** |  | 5.48 (5.18 to 5.78) |
| Education |  | Education |  |
| No qualification | 1 (ref) | No qualification | 1 (ref) |
| Foreign/ other | 0.69 (0.42 to 0.96) *** | Did not finish primary school but can read | 0.81 (0.65 to 0.98)*** |
| NVQ1/CSE or another grade equivalent | 0.47 (0.11 to 0.83) ** | Sishu | 0.32 (-0.48 to 1.11) |
| NVQ2/GCE O Level equivalent | 1.41 (1.21 to 1.61) *** | Elementary school | 1.38 (1.22 to 1.55) *** |
| NVQ3/GCE A Level equivalent | 1.46 (1.17 to 1.74) *** | Middle school | 2.06 (1.88 to 2.23) *** |
| Higher education below degree | 1.67 (1.45 to 1.90) *** | High school | 2.62 (2.37 to 2.87) *** |
| NVQ4/NVQ5/Degree or equivalent | 2.38 (2.15 to 2.61) *** | Vocational school | 3.23 (2.87 to 3.59) *** |
|  |  | Two-three-year college/Associate degree | 4.51 (4.01 to 5.02) *** |
|  |  | Four-year college/Bachelor’s degree | 3.93 (3.25 to 4.60) *** |
| **Rate of linear change** | -0.21 (-0.26 to -0.15) *** | **Rate of linear change** | -0.45 (-0.53 to -0.38) *** |
| Education |  | Education |  |
| No qualification | 1 (ref) | No qualification | 1 (ref) |
| Foreign/ other | 0.009 (-0.04 to 0.05) | Did not finish primary school but can read | 0.15 (0.11 to 0.19) *** |
| NVQ1/CSE or another grade equivalent | -0.02 (-0.08 to 0.04) | Sishu | 0.18 (-0.03 to 0.39) |
| NVQ2/GCE O Level equivalent | 0.02 (-0.01 to 0.06) | Elementary school | 0.26 (0.22 to 0.30) *** |
| NVQ3/GCE A Level equivalent | 0.02 (-0.03 to 0.07) | Middle school | 0.33 (0.28 to 0.37) *** |
| Higher education below degree | 0.03 (-0.009 to 0.07) | High school | 0.36 (0.30 to 0.43) *** |
| NVQ4/NVQ5/Degree or equivalent | 0.01 (-0.02 to 0.05) | Vocational school | 0.30 (0.21 to 0.40) *** |
|  |  | Two-three year college/Associate degree | 0.22 (0.08 to 0.36) *** |
|  |  | Four year college/ Bachelor degree | 0.34 (0.14 to 0.53) *** |
| **Variance ^a^** |  | **Variance ^a^** |  |
| Within-person | 4.97 (4.79 to 5.14) | Within-person | 6.81 (6.66 to 6.97) |
| In initial status | 4.61 (4.32 to 4.93) | In initial status | 2.89 (2.65 to 3.14) |
| In rate of change | 0.04 (0.02 to 0.06) | In rate of change | 0.16 (0.14 to 0.18) |
| **Goodness of fit** |  | **Goodness of fit** |  |
| Deviance (-2LL ^a^) | -63,662.68 | Deviance (-2LL ^a^) | -85,250.51 |
| Wald chi2(29) | 4,019.22 | Wald chi2(29) | 9,396.39 |
| P-value | ≤0.001 | P-value | ≤0.001 |

CI, Confidence Intervals; LL, Log-likelihood; AIC, Akaike’s Information Criterion; BIC, Bayesian Information Criterion. The models include memory, education, covariates (age centred, sex, marital status, heart problems, diabetes, depressive symptoms, alcohol, smoking), time, time ×education, time x baseline memory centred, and time ×covariates (age centred, sex, marital status, heart problems, diabetes, depressive symptoms, alcohol, smoking).

^a^ The within-person variance is the overall residual variance in memory that is not explained by the model. The initial status variance component is the variance of individuals’ intercepts about the intercept of the average person. Likewise, the rate of change variance component is the variance of individual slopes about the slope of the average person.

*p<0.05, **p<0.01, ***p<0.001
